# Cystine transporter SLC7A11/xCT in cancer: ferroptosis, nutrient dependency, and cancer therapy

**DOI:** 10.1007/s13238-020-00789-5

**Published:** 2020-10-01

**Authors:** Pranavi Koppula, Li Zhuang, Boyi Gan

**Affiliations:** 1 Department of Experimental Radiation Oncology, The University of Texas MD Anderson Cancer Center, Houston, TX USA; 2 The University of Texas MD Anderson UTHealth Graduate School of Biomedical Sciences, Houston, TX USA

**Keywords:** SLC7A11, xCT, cystine, cysteine, ferroptosis, nutrient dependency, cancer therapy

## Abstract

The cystine/glutamate antiporter SLC7A11 (also commonly known as xCT) functions to import cystine for glutathione biosynthesis and antioxidant defense and is overexpressed in multiple human cancers. Recent studies revealed that SLC7A11 overexpression promotes tumor growth partly through suppressing ferroptosis, a form of regulated cell death induced by excessive lipid peroxidation. However, cancer cells with high expression of SLC7A11 (SLC7A11^high^) also have to endure the significant cost associated with SLC7A11-mediated metabolic reprogramming, leading to glucose- and glutamine-dependency in SLC7A11^high^ cancer cells, which presents potential metabolic vulnerabilities for therapeutic targeting in SLC7A11^high^ cancer. In this review, we summarize diverse regulatory mechanisms of SLC7A11 in cancer, discuss ferroptosis-dependent and -independent functions of SLC7A11 in promoting tumor development, explore the mechanistic basis of SLC7A11-induced nutrient dependency in cancer cells, and conceptualize therapeutic strategies to target SLC7A11 in cancer treatment. This review will provide the foundation for further understanding SLC7A11 in ferroptosis, nutrient dependency, and tumor biology and for developing novel effective cancer therapies.

## INTRODUCTION

**Figure 1:**
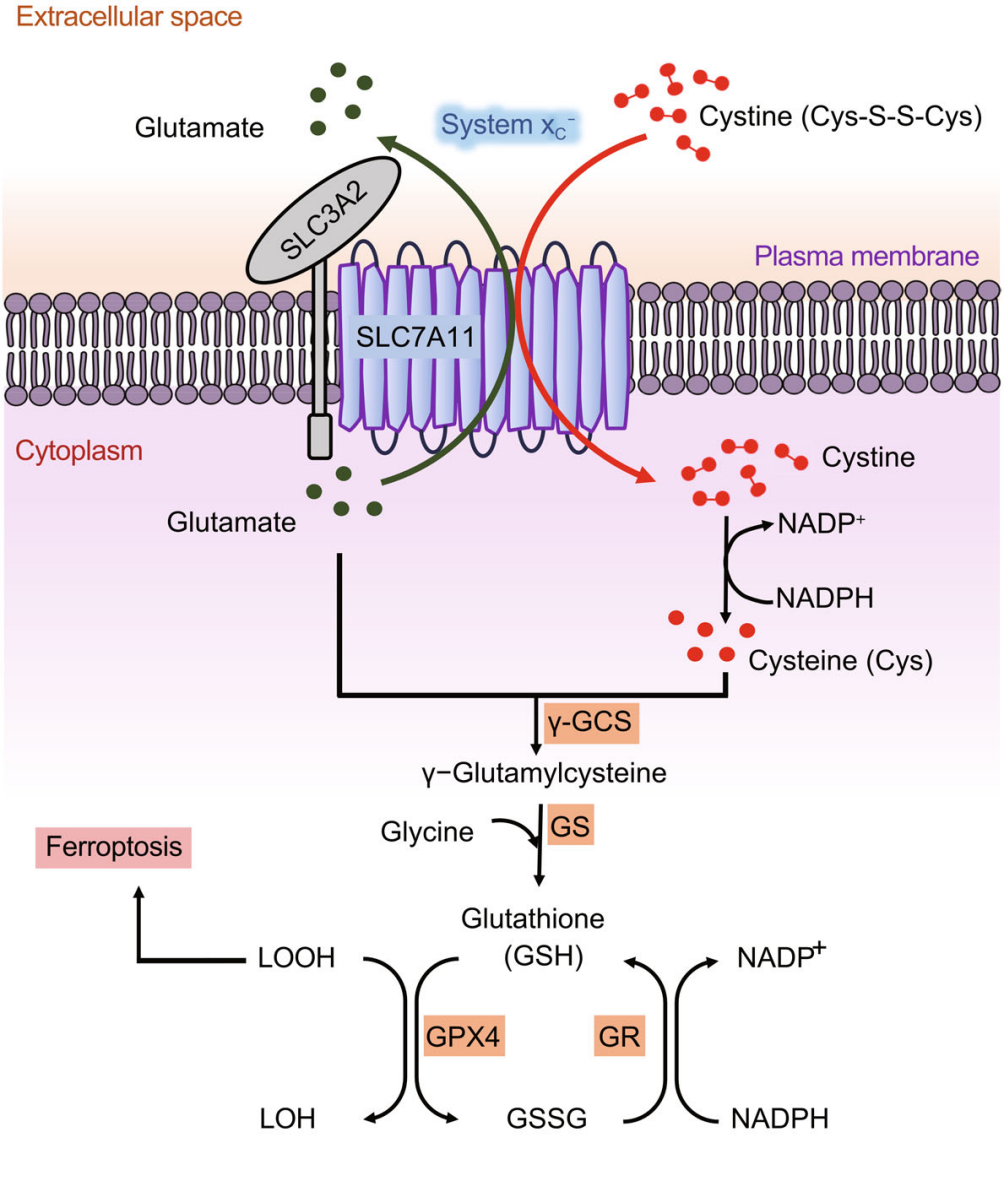
**Structure and function of SLC7A11**. System x_c_^−^ functions as a cystine/glutamate antiporter that imports one molecule of cystine in exchange for one molecule of intracellular glutamate. System x_c_^−^ is a heterodimer consisting of the light chain subunit SLC7A11 and the heavy chain subunit SLC3A2. SLC7A11 mediates the antiporter activity of system x_c_^−^, whereas SLC3A2 anchors SLC7A11 to the plasma membrane and maintains SLC7A11 protein stability. Extracellular cystine is imported into the cell through SLC7A11, and then is converted to cysteine through a NADPH-consuming reduction reaction. Subsequently, cysteine is utilized to synthesize GSH through a two-step process. Cysteine forms γ-glutamylcysteine in conjugation with glutamate in the first step catalyzed by γ-GCS. The second step involves GS-mediated enzymatic addition of a glycine molecule to produce GSH. Ferroptosis is induced by excessive accumulation of lipid hydroperoxides in cellular membrane. GPX4 uses GSH to reduce lipid hydroperoxides to lipid alcohols, thereby suppressing ferroptosis, through which GSH is oxidized to GSSG; GSSG is then converted back to GSH via GR-mediated reduction reaction, which consumes NADPH. γ-GCS: γ-glutamylcysteine synthetase, GS: glutathione synthetase, GPX4: glutathione peroxidase 4, GR: glutathione reductase, GSH: reduced glutathione, GSSG: oxidized glutathione, LOOH: lipid hydroperoxide, LOH: lipid alcohol

Cysteine is a proteinogenic amino acid that has a versatile role in protein synthesis, posttranslational modification, and redox maintenance (Stipanuk et al., 2006; Combs and DeNicola, 2019). Cysteine serves as the rate-limiting precursor for glutathione, a tripeptide comprised of three amino acids—cysteine, glutamate, and glycine—and the most abundant cellular antioxidant. Cysteine can also act as an antioxidant itself as well as the precursor for other biomolecules with antioxidant properties, such as taurine and hydrogen sulfide (Stipanuk et al., 2006; Combs and DeNicola, 2019). Intracellular cysteine can be synthesized through *de novo* biosynthesis (via the transsulfuration pathway) or recycled through protein degradation. However, because cancer cells often experience high levels of oxidative stress (Trachootham et al., 2009; Chio and Tuveson, 2017), cysteine supply via *de novo* biosynthesis or protein catabolism generally cannot meet the high demand for antioxidant defense in cancer cells; therefore, most cancer cells mainly rely on obtaining cysteine from the extracellular environment through nutrient transporters. The intra- and extra-cellular redox environments are very different: while cells generally maintain a reducing environment in the cytosol, the extracellular environment is highly oxidizing. Consequently, extracellular cysteine is highly unstable with only around 30-minute half-life in culture media (Ishii and Bannai, 1985), and is quickly oxidized to cystine (the oxidized dimer form of cysteine). The concentration of extracellular cystine is higher than that of extracellular cysteine by an order of magnitude. Some cancer cells can still effectively take up extracellular cysteine through cysteine transporters under certain conditions; for example, due to the high availability of extracellular cysteine secreted from surrounding bone marrow stromal cells, chronic lymphocytic leukemia cells can actively import cysteine from the extracellular environment (Zhang et al., 2012). However, most cancer cells largely depend on the cystine transporter system x_c_^−^ to import cystine, which is then converted to cysteine in the cytosol through an NADPH-consuming reduction reaction; cysteine is subsequently used to synthesize glutathione (as well as other biomolecules) (Fig. 1) (Stipanuk et al., 2006; Combs and DeNicola, 2019).

The system x_c_^−^ is a sodium-independent antiporter that exports intracellular glutamate and import extracellular cystine at a 1:1 ratio (Fig. 1) (Bannai, 1986; Conrad and Sato, 2012). It consists of two subunits linked via a disulfide bond, including the heavy chain subunit solute carrier family 3 member 2 (SLC3A2; also called CD98 or 4F2hc) and the light chain subunit solute carrier family 7 member 11 (SLC7A11; also commonly known as xCT). SLC7A11, a multi-pass transmembrane protein, mediates the cystine/glutamate antiporter activity in the system x_c_^−^ (Sato et al., 1999; Koppula et al., 2018), whereas SLC3A2, a single transmembrane protein, is a chaperone to maintain SLC7A11 protein stability and appropriate membrane localization (Fig. 1) (Nakamura et al., 1999). Since SLC3A2 also serves as the chaperone for several other amino acid transporters, its function is not limited to cystine transport (Kandasamy et al., 2018). We will focus on SLC7A11 and refer to this cystine transporter as SLC7A11 throughout this review.

The role of cystine uptake in maintaining cell survival was implicated many years ago by the observation that cystine deprivation in cell culture media can lead to significant cell death in many cancer cell lines (Eagle, 1955a, b); however, the nature of this cell death had remained unclear. It was later shown that cystine starvation results in glutathione depletion in cells and that cystine starvation-induced cell death can be rescued by antioxidant vitamin E, therefore indicating the involvement of an oxidative stress-induced cell death in this context (Bannai et al., 1977). In 2012, it was discovered that pharmacologic blockade of SLC7A11-mediated cystine uptake by compounds such as erastin induces a new form of cell death termed ferroptosis (Dixon et al., 2012). Ferroptosis is an iron-dependent form of regulated cell death induced by lethal accumulation of lipid peroxides in the cellular membrane, and is mechanistically and morphologically distinct from other forms of regulated cell death such as apoptosis and necroptosis (Stockwell et al., 2017; Stockwell and Jiang, 2020). Cells have evolved various defense mechanisms to detoxify these toxic lipid peroxides, prominent among which is glutathione peroxidase 4 (GPX4), a GPX family member that uses reduced glutathione (GSH) as a co-factor to detoxify lipid peroxides to lipid alcohols, thereby suppressing ferroptosis (Fig. 1) (Friedmann Angeli et al., 2014; Yang et al., 2014). As noted above, cysteine is the rate-limiting precursor for GSH synthesis, and intracellular cysteine is mainly supplied by SLC7A11-mediated cystine uptake. Consistent with this, it was shown that removing cystine from cell culture media or SLC7A11 inactivation by genetic ablation or pharmacologic inhibition induces potent ferroptosis in many cancer cells; conversely, SLC7A11 overexpression in cancer cells promotes GSH biosynthesis and ferroptosis resistance (Dixon et al., 2012; Jiang et al., 2015; Zhang et al., 2018b). These studies together establish a critical role of SLC7A11-mediated cystine uptake in suppressing ferroptosis and maintaining cell survival under oxidative stress conditions.

In recent years, ferroptosis as a new cell death mechanism has sparked great interest in the scientific community. Accumulating evidence suggests that ferroptosis, similar to apoptosis, is a key tumor suppression mechanism (Jiang et al., 2015; Jennis et al., 2016; Stockwell et al., 2017; Zhang et al., 2018b; Chu et al., 2019; Liu et al., 2019). Importantly, recent studies also revealed that common cancer therapies, such as immunotherapy and radiotherapy, can induce ferroptosis partly through modulating SLC7A11 expression (Lang et al., 2019; Wang et al., 2019a; Lei et al., 2020; Ye et al., 2020). Correspondingly, considerable interest has been directed toward understanding the role and regulatory mechanisms of SLC7A11 in ferroptosis and tumor biology as well as therapeutically targeting SLC7A11 in cancer therapy. Another fascinating topic in SLC7A11 research in recent years has been to uncover the role of SLC7A11 in inducing nutrient dependency in cancer cells (Goji et al., 2017; Koppula et al., 2017; Muir et al., 2017; Romero et al., 2017; Sayin et al., 2017; Shin et al., 2017; Koppula et al., 2018; Joly et al., 2020; Liu et al., 2020). (Here nutrient dependency refers to the phenomenon wherein metabolic reprogramming renders cancer cells to be highly dependent on certain nutrients for survival and growth, such that removal of such nutrients will selectively kill or halt the growth of cancer cells that are dependent on these nutrients while having less harmful effects on other cancer cells or normal cells.) In this review, we will summarize how diverse regulation of SLC7A11 expression and its transporter activity governs ferroptosis. We will then discuss how SLC7A11 promotes tumor development through its function in inhibiting ferroptosis as well as other ferroptosis-independent functions. We will also explore the metabolic underpinnings of SLC7A11-induced nutrient dependency in cancer cells. Finally, we will discuss how our understanding of SLC7A11 function in ferroptosis and nutrient dependency informs strategies to target SLC7A11 in cancer and highlight important unknown questions for future investigation.

## REGULATORY MECHANISMS OF SLC7A11

To ensure appropriate functioning of SLC7A11 in maintaining redox homeostasis, the expression and activity of SLC7A11 are subjected to tight regulation through a variety of mechanisms, including transcriptional regulation by transcription factors and epigenetic regulators, and post-transcriptional regulatory mechanisms to control its mRNA levels, protein stability, subcellular localization, and transporter activity. An emerging theme from recent studies is that various SLC7A11 regulators govern ferroptosis sensitivity through modulating SLC7A11 expression or activity. In this section, we will discuss how different regulatory mechanisms modulate SLC7A11 expression or activity and downstream biological effects mediated by SLC7A11, with a focus on ferroptosis. As further discussed in later sections, some of these SLC7A11 regulators also play important roles in cancer biology, and their dysregulation in cancer can lead to ferroptosis resistance and tumor formation.

### Transcriptional regulation of SLC7A11 by transcriptional factors

**Figure 2:**
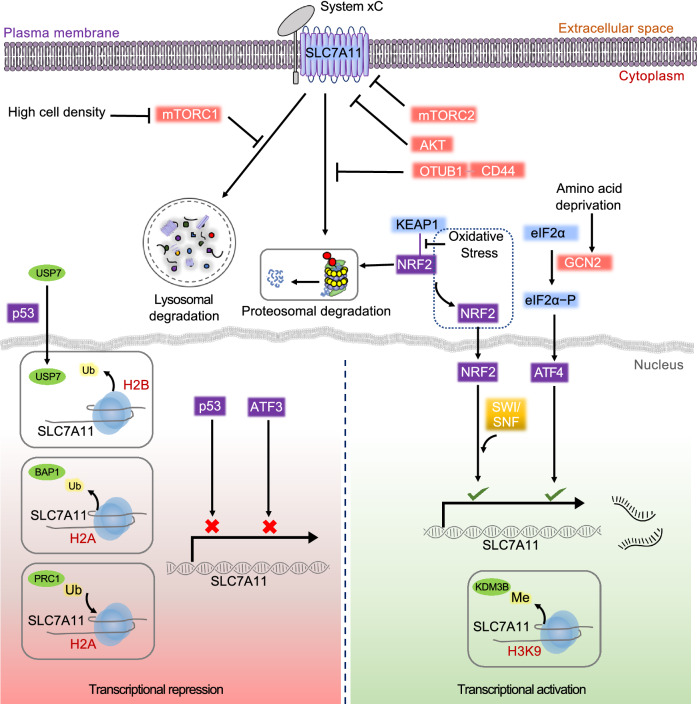
**SLC7A11 regulation by transcriptional, epigenetic, and post-translational mechanisms**. SLC7A11 expression and function can be regulated at multiple levels under basal and stress conditions. *Transcriptional activation*: ATF4 activates *SLC7A11* transcription in response to amino acid deprivation through the GCN2-eIF2α signaling axis. The KEAP1-NRF2 signaling axis regulates oxidative stress-induced *SLC7A11* transcription. In response to oxidative stress, KEAP1-mediated NRF2 proteasomal degradation is suppressed, allowing stabilized NRF2 protein to translocate into the nucleus and activate the transcription of *SLC7A11* and other genes involved in antioxidant response. SWI/SNF chromatin remodeling complexes facilitate NRF2-mediated transcriptional activation of *SLC7A11*. H3K9 demethylase KDM3B decreases H3K9 methylation on the *SLC7A11* promoter and promotes *SLC7A11* transcription. *Transcriptional repression*: Under basal conditions, p53 and ATF3 repress *SLC7A11* transcription. BAP1 deubiquitinates H2Aub on the *SLC7A11* promoter and subsequently represses *SLC7A11*, whereas H2A ubiquitination by PRC1 on the *SLC7A11* promoter also represses *SLC7A11.* p53-mediated nuclear translocation of USP7 results in decreased H2Bub occupancy on the *SLC7A11* promoter via deubiquitination, resulting in transcriptional repression. *Post-translational regulation:* OTUB1 and CD44 form a trimeric complex with SLC7A11 to deubiquitinate SLC7A11 and inhibit SLC7A11 degradation in proteasome, thereby stabilizing SLC7A11 protein, whereas mTORC1 promotes SLC7A11 protein stability by inhibiting its lysosomal degradation. High cell density inhibits mTORC1 and promotes SLC7A11 degradation in lysosomes. Both mTORC2 and AKT inhibit SLC7A11 transporter activity by phosphorylating SLC7A11 at serine 26. ATF4: activating transcription factor 4, GCN2: general control non-derepressible-2, eif2α: eukaryotic initiation factor 2α, KEAP1: Kelch-like ECH associated protein-1, NRF2: nuclear factor erythroid 2-related factor 2, p53: tumor protein p53, ATF3: activating transcription factor 3, BAP1: BRCA1 associated protein-1, PRC1: polycomb repressive complex 1, USP7: ubiquitin-specific-processing protease 7, SWI/SNF: SWItch/sucrose non-fermentable modeling complex, OTUB1: OTU deubiquitinase, ubiquitin aldehyde binding 1, mTORC1: mechanistic target of rapamycin complex 1, mTORC2: mechanistic target of rapamaycin complex 2

It is well established that SLC7A11 expression can be induced under various stress conditions, including oxidative stress, amino acid starvation, metabolic stress, and genotoxic stress, likely as an adaptive response to enable cells to restore redox homeostasis and maintain survival under stress conditions (Koppula et al., 2018). Activating transcription factor 4 (ATF4) and/or nuclear factor erythroid 2-related factor 2 (NRF2) represent two major transcription factors that mediate stress-induced *SLC7A11* transcription. ATF4, a member of the ATF/CREB family of transcription factors, is induced, primarily by mRNA translation, under various stress conditions such as amino acid starvation, endoplasmic reticulum stress, hypoxia, and viral infection; ATF4 then translocates into the nucleus and regulates the transcription of genes that cope with these stress conditions (Pakos-Zebrucka et al., 2016). For example, amino acid deprivation increases *ATF4* mRNA translation through the general control non-derepressible-2 (GCN2)-eukaryotic initiation factor 2α (eIF2α) signaling axis. Consequently, ATF4 binds on the amino acid response elements (AAREs) in gene promoters and promotes the transcription of genes involved in amino acid metabolism and stress response, including *SLC7A11* (Fig. 2), thereby enabling cells to cope with amino acid-limiting conditions (Kilberg et al., 2009). Indeed, *SLC7A11* expression can be potently induced upon deprivation of various amino acids, including cystine, and amino acid deprivation-induced *SLC7A11* expression is largely mediated by ATF4 (Sato et al., 2004). Since cystine starvation induces ferroptosis, whereas SLC7A11 protects cells from ferroptosis (Stockwell and Jiang, 2020), cystine starvation-induced *SLC7A11* expression ostensibly acts as an adaptive response to help cells survive under cystine-limiting conditions. In support of this, it was shown that ATF4 promotes ferroptosis resistance by upregulating SLC7A11 (Chen et al., 2017).

Another transcription factor that promotes *SLC7A11* transcription is NRF2, which primarily mediates transcriptional programs in response to oxidative stress (Rojo de la Vega et al., 2018). NRF2 protein is unstable under basal conditions, being ubiquitinated by an E3 ubiquitin ligase kelch-like ECH-associated protein-1 (KEAP1) and subsequently undergoing rapid degradation through the proteasome pathway. Oxidative stress impairs NRF2 degradation by KEAP1 and stabilizes NRF2 protein, which then binds on the antioxidant response elements (AREs) in gene promoters and governs the transcription of genes involved in antioxidant defense and redox maintenance, including *SLC7A11* (Fig. 2). KEAP1 inactivation leads to NRF2 stabilization under basal conditions, resulting in upregulation of NRF2 target genes (Rojo de la Vega et al., 2018). Recent studies using clustered regularly interspaced short palindromic repeats (CRISPR) screens uncovered KEAP1 as a key regulator of ferroptosis, and it was shown that *KEAP1* deficiency promotes ferroptosis resistance (Cao et al., 2019). Considering the critical role of SLC7A11 in inhibiting ferroptosis, it is likely that the ferroptosis resistance caused by *KEAP1* deficiency is at least partly mediated by SLC7A11. It should be noted that NRF2 regulates other genes involved in GSH biosynthesis, iron metabolism, and antioxidant responses, which might also play a role in mediating ferroptosis resistance in *KEAP1* deficient cells. An extensive discussion of this topic will be beyond the scope of this review, and we refer readers to an excellent recent review focusing on NRF2 signaling in ferroptosis (Anandhan et al., 2020).

ATF4 and NRF2 often act concertedly to regulate stress-induced gene expression. For example, ATF4 and NRF2 interact with each other on the *SLC7A11* promoter, and cooperatively regulate *SLC7A11* transcription under stress conditions (Ye et al., 2014). It is important to note that the definitive evidence for the requirement of ATF4 or NRF2 to mediate various stress-induced *SLC7A1*1 expression is often lacking (i.e., to show that *ATF4* or *NRF2* deletion would abolish SLC7A11 induction under a given stress condition). A recent study showed that *ATF4* or *NRF*2 deficiency abolished glucose starvation-induced *SLC7A1*1 expression; surprisingly, erastin-induced SLC7A11 remains intact in *ATF4* or *NRF2* knockout (KO) cells (Zhang et al., 2019a), suggesting that while glucose starvation-induced *SLC7A11* expression depends on ATF4 and NRF2, erastin-induced *SLC7A11* expression likely operates through ATF4- or NRF2-independent mechanisms.

*SLC7A11* expression can also be repressed by transcriptional regulation. p53 is a transcription factor that plays a key role in tumor suppression (Vousden and Prives, 2009). *SLC7A11* was identified as a p53 transcriptional target that is repressed by p53 (Fig. 2) (Jiang et al., 2015). It was further shown that p53 promotes ferroptosis under various ferroptosis-inducing conditions partly by repressing *SLC7A11* expression, whereas SLC7A11 upregulation by *p53* deficiency promotes ferroptosis resistance (Jiang et al., 2015). As further elaborated in the following section, p53 regulation of ferroptosis via suppressing *SLC7A11* expression plays a key role in tumor suppression.

ATF3, another member of the ATF/CREB family of transcription factors, was previously identified to be upregulated upon erastin treatment (Dixon et al., 2014). A subsequent study confirmed erastin-induced ATF3 expression and further showed that ATF3 can bind on the *SLC7A11* promoter and repress its expression independent of p53 (Fig. 2), and ATF3 promotes erastin-induced ferroptosis via downregulating SLC7A11 levels (Wang et al., 2020a). Since erastin treatment (or cystine starvation) potently induces *SLC7A11* expression (Zhang et al., 2018b) yet ATF3 suppresses *SLC7A11* expression (Wang et al., 2020a), it seems unlikely that erastin-induced ATF3 would contribute to erastin-induced *SLC7A11* expression; it was further demonstrated that erastin-induced ATF3 does not appear to regulate SLC7A11 levels induced by erastin treatment (Wang et al., 2020a). These data suggest that ATF3 primarily regulates *SLC7A11* transcription at basal conditions and does not affect stress-induced *SLC7A11* expression even though ATF3 is often induced upon stress. In brief summary, various stress conditions promote *SLC7A11* transcription partly through ATF4 and/or NRF2, whereas p53 and ATF3 mainly repress *SLC7A11* expression under basal conditions (Fig. 2). Consequently, regulation of *SLC7A11* expression by these transcription factors leads to altered cellular sensitivity to ferroptosis.

### Epigenetic regulation of SLC7A11 transcription

Epigenetic regulation of gene transcription is primarily achieved by chemical modifications on DNA (such as DNA methylation) and/or DNA-associated histones (such as histone acetylation, methylation, ubiquitination, and phosphorylation) (Jaenisch and Bird, 2003). These modifications are added, removed, and recognized by various epigenetic regulators collectively called writers, erasers, and readers, respectively, resulting in transcriptional activation or repression (Badeaux and Shi, 2013). Specific DNA or histone modifications are often associated with specific types of gene transcription. For example, mono- and tri-methylation at lysine 4 of histone H3 (H3K4me1 and H3K4me3) are associated with transcriptional activation, whereas tri-methylation at lysine 9 and lysine 27 of histone H3 (H3K9me3 and H3K27me3) correlate with transcriptional repression. Epigenetic regulation of gene transcription is critical for controlling cellular homeostasis and development, and its dysregulation results in diseases such as cancer. Recent studies have revealed a critical role of epigenetic regulation of *SLC7A11* transcription in governing ferroptosis.

BAP1 is a nuclear deubiquitinase (DUB) that removes histone 2A mono-ubiquitination (H2Aub) at lysine 119, a histone modification that is generally associated with transcriptional repression (Wang et al., 2004; Scheuermann et al., 2010). BAP1 and its interacting proteins form the polycomb repressive deubiquitinase (PR-DUB) complex (Carbone et al., 2013). In a recent study, genome-wide analyses identified SLC7A11 as a key transcriptional target of BAP1 (Zhang et al., 2018b). Mechanistically, the PR-DUB complex binds on the *SLC7A11* promoter, allowing BAP1 to deubiquitinate H2Aub on the *SLC7A11* promoter and subsequently repress *SLC7A11* expression independent of p53 (Fig. 2), resulting in decreased cystine uptake and increased ferroptosis sensitivity; conversely, *BAP1* deficiency in cancer cells leads to *SLC7A11* upregulation and ferroptosis resistance (Zhang et al., 2018b; Zhang et al., 2019c).

Based on the current model that H2Aub is associated with gene repression (Scheuermann et al., 2010), it would be expected that BAP1, by removing H2Aub, should mainly activate gene transcription. Therefore, the finding that BAP1 represses *SLC7A11* expression seems counterintuitive. However, genome-wide analyses showed that BAP1-mediated deubiquitination of H2Aub is associated with both transcriptional activation and repression of target genes (Zhang et al., 2018b; Artegiani et al., 2019). These data raised the question of how BAP1-mediated deubiquitination of H2Aub regulates transcriptional repression. Polycomb repressive complex 1 (PRC1) is a major ubiquitin ligase to mediate H2Aub (Wang et al., 2004). It was shown that, while PRC1 and BAP1 (the writer and eraser of H2Aub, respectively) exert opposing effects on H2Aub levels on the *SLC7A11* promoter (i.e., PRC1 inactivation decreases whereas BAP1 inactivation increases H2Aub levels on the *SLC7A11* promoter), both PRC1 and BAP1 repress *SLC7A11* expression (Fig. 2) (Zhang et al., 2019a). These data are in line with previous observations showing that inactivation of *Drosophila* PRC1 or BAP1 leads to upregulation of HOX genes (Scheuermann et al., 2010), and suggest a model that a dynamic balance between H2A ubiquitination and deubiquitination by PRC1 and BAP1, rather than H2Aub *per se*, might be important for repressing the expression of certain target genes such as SLC7A11 and HOX genes.

While H2Aub is associated with transcriptional repression, mono-ubiquitination of histone 2B (H2Bub) at lysine 120 generally correlates with transcriptional activation (Henry et al., 2003). A recent study linked H2Bub-mediated epigenetic mechanisms to transcriptional activation of *SLC7A11* and ferroptosis suppression (Wang et al., 2019b). It was shown that erastin treatment decreases global H2Bub levels as well as H2Bub occupancy on the *SLC7A11* promoter, resulting in *SLC7A11* transcriptional repression (Fig. 2) (Wang et al., 2019b). [As noted above, erastin treatment generally increases *SLC7A11* expression. These data suggest that erastin treatment can induce two opposing effects on *SLC7A11* transcription: transcriptional activation (through a still unknown mechanism) and transcriptional repression (through decreasing H2Bub occupancy on the *SLC7A11* promoter (Wang et al., 2019b) and potentially activating ATF3 (Wang et al., 2020a)]. It seems that erastin-induced *SLC7A11* transcriptional activation overrides its transcriptional repression, resulting in increased *SLC7A11* expression as a net effect.] It was further demonstrated that a reduction of H2Bub levels upon erastin treatment promotes ferroptosis sensitivity, possibly through repressing *SLC7A11* expression. Mechanistically, it was proposed that p53 promotes the nuclear translocation of USP7, a DUB to remove ubiquitin from H2Bub, resulting in decreased H2Bub occupancy on the *SLC7A11* promoter and subsequent *SLC7A11* transcriptional repression (Fig. 2) (Wang et al., 2019b).

H3K9 methylation has been shown to correlate with transcriptional repression (Stewart et al., 2005). It was recently reported that overexpression of KDM3B, a H3K9 demethylase, decreases H3K9 methylation and upregulates *SLC7A11* expression (Fig. 2), presumably by removing this repressive histone mark on the *SLC7A11* promoter, resulting in enhanced resistance to erastin-induced ferroptosis (Wang et al., 2020b). As another example, BRD4, a member of the bromodomain and extraterminal domain (BET) family of proteins, is an important reader protein capable of recognizing acetylated histones and recruiting transcription factors to regulate gene transcription. Pharmacological inhibition of BRD4 by its inhibitors such as JQ1 represents a promising therapeutic strategy in cancer treatment (Shi and Vakoc, 2014). A recent study showed that JQ1 can induce ferroptosis both in vitro and in xenograft tumors; furthermore, JQ1 treatment or *BRD4* knockdown results in downregulation of SLC7A11 as well as other ferroptosis regulators (Sui et al., 2019), suggesting that BRD4 might activate *SLC7A11* transcription via epigenetic mechanisms, whereas JQ1-mediated BRD4 inhibition represses *SLC7A11* expression and promotes ferroptosis.

Another critical epigenetic mechanism to control gene transcription involves chromatin remodeling mediated by SWI/SNF complexes, which govern histone insertion, ejection, and nucleosome sliding and therefore control gene transcription (Kadoch and Crabtree, 2015). SWI/SNF complex-mediated chromatin remodeling was recently implicated to regulate *SLC7A11* transcription (Ogiwara et al., 2019). It was shown that ARID1A, an integral component of SWI/SNF complexes, binds on the *SLC7A11* promoter and facilitates NRF2-mediated transcriptional activation of *SLC7A11* through chromatin remodeling by SWI/SNF complexes (Fig. 2); *ARID1A* deficiency results in *SLC7A11* transcriptional repression, impaired cystine uptake and GSH biosynthesis, and subsequently ROS induction (Ogiwara et al., 2019). Together, these recent studies highlight critical roles of histone modifications and chromatin remodeling in governing *SLC7A11* expression and ferroptosis.

### Posttranslational regulation of SLC7A11

Apart from transcriptional regulation as discussed above, *SLC7A11* expression can also be regulated at mRNA levels by nonsense-mediated mRNA decay and microRNAs (Liu et al., 2011; Drayton et al., 2014; Martin and Gardner, 2015; Wu et al., 2017), the detailed mechanisms of which have been discussed in a previous review (Koppula et al., 2018). Below we focus on various aspects of posttranslational regulation of SLC7A11 as revealed by more recent studies.

Earlier studies identified adhesion molecule CD44 variant (CD44v) as a SLC7A11-binding partner that regulates SLC7A11 protein stability, and showed that CD44v inactivation leads to decreased stability and defective cell surface localization of SLC7A11, resulting in compromised SLC7A11 function in regulating GSH synthesis, redox maintenance, and tumor growth and metastasis (Ishimoto et al., 2011; Yae et al., 2012). Since CD44v is neither a ubiquitin ligase nor a DUB, how VD44v regulates SLC7A11 protein stability had remained elusive. A recent study sheds light on this question. OTUB1, a DUB of the ovarian tumor (OTU) family, was identified as a SLC7A11-interacting protein which stabilizes SLC7A11 by preventing SLC7A11 from undergoing ubiquitination and proteasomal degradation (Fig. 2) (Gan, 2019; Liu et al., 2019). It was further shown that OTUB1, CD44v, and SLC7A11 form a trimeric complex and that CD44v promotes SLC7A11 protein stability by maintaining the interaction between OTUB1 and SLC7A11; consequently, inactivation of CD44v or OTUB1 destabilizes SLC7A11 and increases ferroptosis sensitivity (Liu et al., 2019). The potential ubiquitin ligase which mediates SLC7A11 ubiquitination and proteasomal degradation remains to be identified.

Recent evidence also indicated that, as a transmembrane protein localized on the plasma membrane, SLC7A11 is also subjected to regulation by lysosomal degradation. As detailed in a later section, SLC7A11 promotes glucose dependency, leading to the exquisite vulnerability of cancer cells with high SLC7A11 expression (SLC7A11^high^) to glucose starvation (Goji et al., 2017; Koppula et al., 2017; Shin et al., 2017; Joly et al., 2020; Liu et al., 2020). It is known that cells cultured at high density survive much better than those cultured at low density under glucose starvation. A recent study showed that the better survival of cancer cells under higher density is at least partly achieved by downregulation of SLC7A11 levels (Yamaguchi et al., 2020). Mechanistically, it was proposed that higher cell density inactivates mechanistic target of rapamycin complex 1 (mTORC1), resulting in SLC7A11 degradation in lysosomes (Fig. 2). Although how cell density modulates mTORC1 activity was not revealed in this study, another recent study showed that high cell density activates LATS1/2 kinases, a key component in the Hippo pathway, which then phosphorylates Raptor, an integral component of mTORC1, resulting in mTORC1 inactivation (Gan et al., 2020). How mTORC1 inactivation subsequently promotes SLC7A11 lysosomal degradation remains unknown currently.

mTOR exists in two distinct kinase complexes, mTORC1 and mTOR complex 2 (mTORC2). While mTORC1 coordinates nutrient and energy availability with protein synthesis and cell growth, mTORC2 integrates growth factor signaling to promote cell proliferation and survival mainly through phosphorylating AKT (Kim and Guan, 2019). Notably, SLC7A11 can be regulated not only by mTORC1 (Gan et al., 2020), but also by mTORC2 (Gu et al., 2017). It was shown that mTORC2 interacts with SLC7A11 and phosphorylates serine 26 located at the cytoplasmic region of SLC7A11 in response to growth factor stimulation, resulting in inhibition of its transporter activity (Fig. 2) (Gu et al., 2017). Interestingly, another study revealed that AKT, a major substrate of mTORC2, is capable of directly phosphorylating SLC7A11 on the same site, and AKT-mediated SLC7A11 phosphorylation also inhibits its cystine transport activity (Fig. 2) (Lien et al., 2017).

Other studies revealed that cell surface localization of SLC7A11 is also subjected to regulation. EGF receptor (EGFR) was shown to interact with SLC7A11 and maintain its appropriate localization on the plasma membrane (Tsuchihashi et al., 2016). Consequently, EGFR-expressing glioma cells exhibit increased cystine uptake and GSH biosynthesis as well as enhanced glutamate export, which together promote tumor growth and invasion (Tsuchihashi et al., 2016). Together, these emerging studies established that SLC7A11 can be regulated by diverse posttranslational mechanisms, including regulation of protein stability, localization, and transporter activity.

## SLC7A11 PROMOTES TUMOR DEVELOPMENT PARTLY VIA INHIBITING FERROPTOSIS

It is well established that cell death, most notably apoptosis, plays important roles in tumor suppression (Green and Evan, 2002; Igney and Krammer, 2002), and cell death resistance is a hallmark of cancer (Hanahan and Weinberg, 2011). Recent studies have unraveled that ferroptosis, similar to apoptosis, represents another powerful tumor suppression mechanism, which presumably has evolved to eliminate precancerous cells that have been exposed to metabolic stress or nutrient deprivation. Importantly, SLC7A11 has emerged as a central hub linking ferroptosis to its proposed tumor suppression function. In this section, we will discuss how SLC7A11-mediated ferroptosis suppression contributes to tumor development in the context of several tumor suppressors and oncogene pathways.

**Figure 3:**
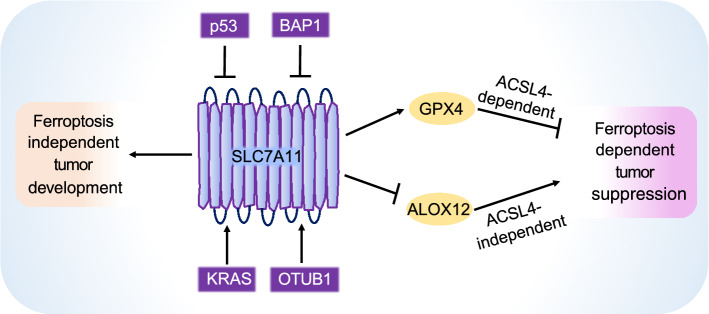
**SLC7A11 promotes tumor development through both ferroptosis-dependent and -independent mechanisms**. SLC7A11 promotes tumor development partly through detoxifying lipid peroxides and suppressing ferroptosis, which involves both ASCL4-dependent (via GPX4) and -independent (via ALOX12) pathways. Tumor suppressor proteins such as p53 and BAP1 inhibit tumorigenesis partly by suppressing SLC7A11 expression and promoting ferroptosis, while oncogenic KRAS and OTUB1 promote tumor development partly by promoting SLC7A11 levels and inhibiting ferroptosis. SLC7A11 can also promote tumorigenesis via pathways that are not dependent on its function to suppress ferroptosis. KRAS: Kirsten rat sarcoma viral oncogene homolog, OTUB1: OTU deubiquitinase, ubiquitin aldehyde binding 1, p53: tumor protein p53, BAP1: BRCA1 associated protein-1, ACSL4: Acyl-CoA synthetase long chain family member 4, GPX4: glutathione peroxidase 4, ALOX12: arachidonate 12-lipoxygenase

*p53* is the most mutated tumor suppressor in human cancers (Muller and Vousden, 2013). Our mechanistic understanding of p53 function in tumor biology has provided great insights into the role of ferroptosis in tumor suppression. Traditionally, the tumor-suppressive function of p53 has been largely attributed to its roles in inducing cell cycle arrest, senescence, and/or apoptosis. Curiously, a p53 mutant that cannot be acetylated on certain lysine residues (3KR mutant) loses its ability to induce cell cycle arrest, senescence, or apoptosis, yet is still capable of suppressing tumor formation *in vivo* (Li et al., 2012). This p53 mutant was later discovered to retain its tumor-suppressive function, at least partly by repressing *SLC7A11* expression and inducing ferroptosis (Jiang et al., 2015). Mutation of an additional acetylation site at the backdrop of p53 3KR mutant markedly attenuated p53’s capability to suppress tumor formation, repress *SLC7A11* expression, and induce ferroptosis in cancer cells (Wang et al., 2016). An African-specific polymorphic mutant of p53 (S47 mutant) provides another evidence to link p53 regulation of SLC7A11 and ferroptosis to tumor suppression. Opposite to p53 3KR mutant, p53 S47 mutant retains its capability to induce cell cycle arrest and apoptosis, yet is unable to suppress tumor development (Jennis et al., 2016). It was further shown that this mutant exhibits impaired ability to repress SLC7A11 expression or induce ferroptosis, suggesting that SLC7A11 downregulation and ferroptosis induction play a role in p53-mediated tumor suppression. Collectively, these studies using different p53 mutants suggest that ferroptosis induction caused by *SLC7A11* repression at least partly underlies p53 function in tumor suppression (Fig. 3).

It is well established that SLC7A11 inhibits ferroptosis through importing cystine, promoting GSH biosynthesis, and subsequently facilitating GPX4-mediated detoxification of lipid peroxides (Fig. 1) (Stockwell et al., 2017). Ferroptosis induced by SLC7A11 or GPX4 inactivation can be largely abolished by inactivation of ACSL4, a lipid metabolism enzyme (Doll et al., 2017; Kagan et al., 2017; Stockwell and Jiang, 2020). Interestingly, a recent study proposed that SLC7A11 suppression downstream of p53 promotes ferroptosis through GSH-GPX4- and ACSL4-independent mechanisms (Chu et al., 2019). Mechanistically, it was shown that SLC7A11 interacts with arachidonate 12-lipoxygenase (ALOX12), and this interaction suppresses ALOX12’s lipoxygenase activity to mediate polyunsaturated fatty acid (PUFA) peroxidation, thereby inhibiting ferroptosis; consequently, inactivation of ALOX12, but not ACSL4, abrogates p53-mediated ferroptosis (Chu et al., 2019). Importantly, *ALOX12* gene is frequently deleted in human cancers (Chu et al., 2019). It was further demonstrated that deletion of one *Alox12* allele in mouse promotes lymphomagenesis in Eμ-Myc models, and that *ALOX12* missense mutations derived from human cancers lose their capabilities to oxygenate PUFA and to promote p53-mediated ferroptosis (Chu et al., 2019). This study suggests that, in the context of *p53* deficiency, SLC7A11 can promote tumor development through a non-canonical mechanism by directly interacting with ALOX12 and inhibiting ALOX12-mediated ferroptosis, which seems to be independent of its cystine transporting activity (Fig. 3).

*BAP1* is another tumor suppressor that is frequently mutated or deleted in several human cancers (Carbone et al., 2013). As discussed above, recent studies identified SLC7A11 as a key downstream target of BAP1 (Zhang et al., 2018b). Functional analyses revealed that, by suppressing *SLC7A11* transcription, BAP1 inhibits cystine uptake and GSH biosynthesis and promotes ferroptosis under various ferroptosis-inducing conditions; furthermore, tumor growth suppression caused by BAP1 restoration in *BAP1*-deficient tumors can be partly abolished by SLC7A11 overexpression or treatment with ferroptosis inhibitor, and *BAP1* mutations derived from human cancers abrogate their abilities to suppress *SLC7A11* expression and to induce ferroptosis (Zhang et al., 2018b). Therefore, similar to p53, BAP1 suppresses tumor development at least partly by inducing ferroptosis upon SLC7A11 repression (Fig. 3) (Zhang et al., 2019c). However, unlike p53, it seems that BAP1 promotes ferroptosis through the canonical function of SLC7A11 (i.e., through SLC7A11-mediated cystine uptake to promote GSH biosynthesis). Since both p53 and BAP1 regulate SLC7A11 at the transcriptional level, it remains unclear how SLC7A11 shifts between its canonical and non-canonical functions in ferroptosis regulation in these two different contexts.

In cancer cells, not only de-repression of *SLC7A11* expression, but also stabilization of SLC7A11 protein can lead to enhanced tumor formation through inhibiting ferroptosis. As discussed in the previous section, OTUB1 deubiquitinates and stabilizes SLC7A11 protein (Liu et al., 2019). Consequently, OTUB1 promotes tumor development partly via stabilizing SLC7A11 and inhibiting ferroptosis (Fig. 3) (Liu et al., 2019). Since OTUB1 is frequently overexpressed in human cancers, high SLC7A11 levels in some cancers might result from post-translational regulation by OTUB1.

Emerging data suggest that SLC7A11-mediated ferroptosis inhibition not only plays a role in tumor development caused by loss of tumor suppressors (such as *p53* and *BAP1*), but also contributes to oncogene-driven tumorigenesis. *KRAS* is one of the most mutated proto-oncogene in human cancers, and is highly mutated in a variety of cancers, including pancreatic ductal adenocarcinomas (PDAC), lung cancer, and colorectal cancer (Prior et al., 2012). Recent studies showed that oncogenic KRAS promotes *SLC7A11* transcription, resulting in increased SLC7A11-mediated cystine uptake and GSH biosynthesis (Fig. 3) (Lim et al., 2019; Hu et al., 2020). Mechanistically, one study suggested that ETS-1 transcription factor mediates oncogenic KRAS-induced *SLC7A11* expression via cooperation with ATF4 (Lim et al., 2019), while the other study indicated that oncogenic KRAS promotes *SLC7A11* transcription through NRF2 (Hu et al., 2020). It was further shown that genetic ablation or pharmacological inhibition of SLC7A11 markedly attenuates oncogenic KRAS-induced tumor growth in xenograft models (Lim et al., 2019; Hu et al., 2020). More than 90% of PDAC harbor *KRAS* mutations (Prior et al., 2012). SLC7A11 inactivation, genetically or pharmacologically, was shown to potently induce lipid peroxidation and ferroptotic cell death in pancreatic cancer cells (Daher et al., 2019; Badgley et al., 2020); further studies using genetically engineered mouse models (GEMMs) demonstrated that deletion of *Slc7a11* significantly suppresses oncogenic *Kras*-driven PDAC development with ferroptosis induction in *Slc7a11*-deficient pancreatic tumors with oncogenic Kras activation (Badgley et al., 2020), suggesting that SLC7A11-mediated ferroptosis suppression likely contributes to KRAS-driven PDAC development.

It should be noted that not all SLC7A11 expression alterations in cancers can be linked to its presumed function to mitigate ferroptosis and promote tumor development. Tumor suppressor *ARID1A* is frequently mutated in multiple forms of cancers (Jones et al., 2010; Mao and Shih Ie, 2013). As discussed in the previous section, ARID1A-containing SWI/SNF complex promotes *SLC7A11* transcription. In contrast to *p53* or *BAP1* deficiency (wherein tumor suppressor deficiency increases *SLC7A11* expression), *ARID1A* deficiency suppresses *SLC7A11* expression in cancer cells (Ogiwara et al., 2019). It was further shown that the suppressed *SLC7A11* expression results in decreased cystine uptake and GSH biosynthesis in *ARID1A*-deficient cancer cells, which induces a vulnerability rendering *ARID1A*-deficient cancers susceptible for pharmacological inhibition of GSH biosynthesis (Ogiwara et al., 2019). Tumors with *p53* gain-of-function mutations present another example wherein tumors actually exhibit reduced SLC7A11 levels. While many tumors harbor *p53* loss-of-function mutations or deletions, others contain oncogenic gain-of-function mutations of *p53*, resulting in the expression of mutant p53 protein in tumors, and such tumors often exhibit poor prognosis and aggressive tumor phenotypes (Muller and Vousden, 2013). Based on the findings that wild-type (WT) p53 represses *SLC7A11* expression and *p53* deficiency promotes *SLC7A11* expression in cancer cells (Jiang et al., 2015), one would expect that p53 mutants with oncogenic functions, similar to *p53* deficiency, should promote *SLC7A11* expression. In contrast, it was demonstrated that p53 mutants somehow gain additional functional capabilities to inhibit NRF2-mediated transcriptional activation of *SLC7A11*, resulting in decreased levels of SLC7A11 and GSH and increased ROS and lipid peroxidation in *p53* mutant cancer cells, and rendering such tumors more sensitive to SLC7A11 inhibitors such as sulfasalazine (Liu et al., 2017). Whether NRF2 is involved in WT p53-mediated *SLC7A11* repression remains to be examined.

To summarize, consistent with the known role of SLC7A11 in suppressing ferroptosis and the emerging concept that ferroptosis is a tumor suppression mechanism, various studies have revealed that loss of tumor suppressors (such as *p53* and *BAP1*) (Jiang et al., 2015; Zhang et al., 2018b), mutation of proto-oncogenes (such as *KRAS*) (Badgley et al., 2020), or overexpression of proteins with pro-tumorigenic functions (such as OTUB1) (Liu et al., 2019) increases SLC7A11 levels in cancer (by either upregulating its transcription or stabilizing its protein), resulting in ferroptosis suppression and enhanced tumor development (Fig. 3). However, not all tumors show increased SLC7A11 levels, and some tumors (such as *ARID1A*-deficient tumors or tumors with *p53* gain-of-function mutations) even exhibit decreased SLC7A11 levels (Liu et al., 2017; Ogiwara et al., 2019). In this latter scenario, the decreased SLC7A11 levels apparently cannot explain tumor phenotypes in the corresponding cancers; instead, it is proposed that this decreased SLC7A11 expression creates an Achilles heel for therapeutic targeting in tumors with low expression of SLC7A11, as such tumors presumably should be more vulnerable to oxidative stress.

## FERROPTOSIS-INDEPENDENT FUNCTIONS OF SLC7A11 IN PROMOTING TUMOR DEVELOPMENT

Although this review so far has somewhat focused on the anti-ferroptosis function of SLC7A11 in tumor biology (partly because ferroptosis is a relatively recently discovered cell death mechanism), we want to emphasize that SLC7A11’s roles in tumor biology clearly go beyond regulating ferroptosis. In this section, we also discuss several other functions of SLC7A11 which likely operate independent of ferroptosis (Fig. 3).

While SLC7A11’s function in suppressing ferroptosis is well established, other studies have shown that SLC7A11 inactivation can also induce apoptosis. It was initially shown that *Slc7a11* deficiency in mouse melanocytes induces substantial cell death accompanied with features of apoptosis such as caspase-3 cleavage (Qiao et al., 2008). Subsequent studies revealed that SLC7A11 inhibition can induce apoptosis in cancer cells under different contexts (Liu et al., 2011; Yoshikawa et al., 2013; Dai et al., 2014). While erastin potently induces ferroptosis by inhibiting SLC7A11-mediated cystine uptake (Stockwell et al., 2017), HG106, another recently identified SLC7A11 inhibitor, does not induce ferroptosis but mainly induces apoptosis (Hu et al., 2020). Since these studies suggested that SLC7A11 inactivation-induced apoptosis is likely driven by GSH depletion and ROS induction, it remains to be established how GSH depletion resulting from SLC7A11 inactivation can induce different modes of cell death (ferroptosis vs apoptosis) under different conditions or contexts. The differential effects of erastin and HG106 may relate to their different potencies or off-target effects as well as different cell lines used in these studies (such that SLC7A11 inactivation results in ferroptosis in one cell line but induces apoptosis in another cell line). Future studies using respective cell death inhibitors or cell death deficient cells (such as *BAX*/*BAK* or *ACSL4* KO cells) are required to address these questions. Furthermore, a side-by-side comparison between erastin and HG106 in the same cell lines will be critical to clarify the differential cell death-inducing effects of these SLC7A11 inhibitors.

In addition, inhibition of SLC7A11, genetically or pharmacologically, has been shown to result in cellular defects typically associated with oncogenic pathway inactivation, including suppression of cell proliferation/growth and invasion, defective clonogenic survival, and decreased anchorage-independent growth, whereas SLC7A11 overexpression can exert the opposite cellular effects (Ishimoto et al., 2011; Yae et al., 2012; Yang and Yee, 2014; Ji et al., 2018; Shin et al., 2018; Hu et al., 2020). Although the underlying mechanisms are often not definitively established, SLC7A11’s ability to promote GSH biosynthesis has been attributed to regulate these cellular functions. In another study, SLC7A11-mediated glutamate export has been linked to the increased invasiveness of breast cancer cells (Dornier et al., 2017).

As noted above, various stress conditions induce *SLC7A11* expression to promote cellular adaption to stress conditions. Correspondingly, it is well established that SLC7A11 promotes drug-, chemo-, and radio-resistance in cancer cells. SLC7A11 overexpression has been shown to correlate with or functionally promote resistance to various therapeutic drugs, such as cisplatin, gemcitabine, and MAPK pathway inhibitors (Okuno et al., 2003; Lo et al., 2008; Wang et al., 2018). SLC7A11 also confers resistance to geldanamycin, thereby promoting chemoresistance (Huang et al., 2005). Finally, it was shown that radiation can induce *SLC7A11* expression (Xie et al., 2011; Lei et al., 2020), and SLC7A11 overexpression promotes radioresistance, whereas SLC7A11 inhibition enhances radiosensitivity in cancer cells or tumors (Cobler et al., 2018; Nagane et al., 2018; Lei et al., 2020); importantly, the radioresistance caused by SLC7A11 overexpression was largely abolished under ferroptosis inhibitor treatment (Lei et al., 2020), suggesting that SLC7A11 promotes radioresistance mainly through inhibiting ferroptosis. It remains to be determined whether ferroptosis inhibition also plays a role in SLC7A11-mediated drug- or chemo-resistance, although it is generally believed that SLC7A11 promotes these resistance phenotypes through enhancing GSH synthesis.

Most of the cellular functions we have discussed so far relate to cell autonomous effects regulated by SLC7A11. Emerging evidence indicated that SLC7A11 can also exert cell non-autonomous effects on tumor microenvironment through glutamate exporting. A recent study revealed that treatment with VEGF blockade agents in a glioma mouse model leads to increased expression of *SLC7A11*, and it was proposed that glutamate exported by SLC7A11 from tumor cells then promotes the proliferation and immunosuppressive function of regulatory T-cells (Tregs), which contributes to adaptive resistance to anti-VEGF therapy in glioblastoma (Long et al., 2020). It was further shown that blocking Tregs using anti-CD25 antibody could sensitize tumors to VEGF blockade agents (Long et al., 2020). While most studies have focused on SLC7A11-mediated cystine import, this study suggests that SLC7A11-mediated glutamate export can cross-talk with and modulate tumor immune systems, which in turn influence tumor growth and response to cancer therapies.

In summary, ferroptosis-independent functions of SLC7A11 in tumor biology include its regulation of other non-ferroptotic cell death, cell proliferation, cell invasion, chemo-/drug-/radio-resistance, and tumor immunity, which can be achieved through SLC7A11-mediated cystine import and/or glutamate export.

## SLC7A11 INDUCES NUTRIENT DEPENDENCY IN CANCER CELLS

**Figure 4:**
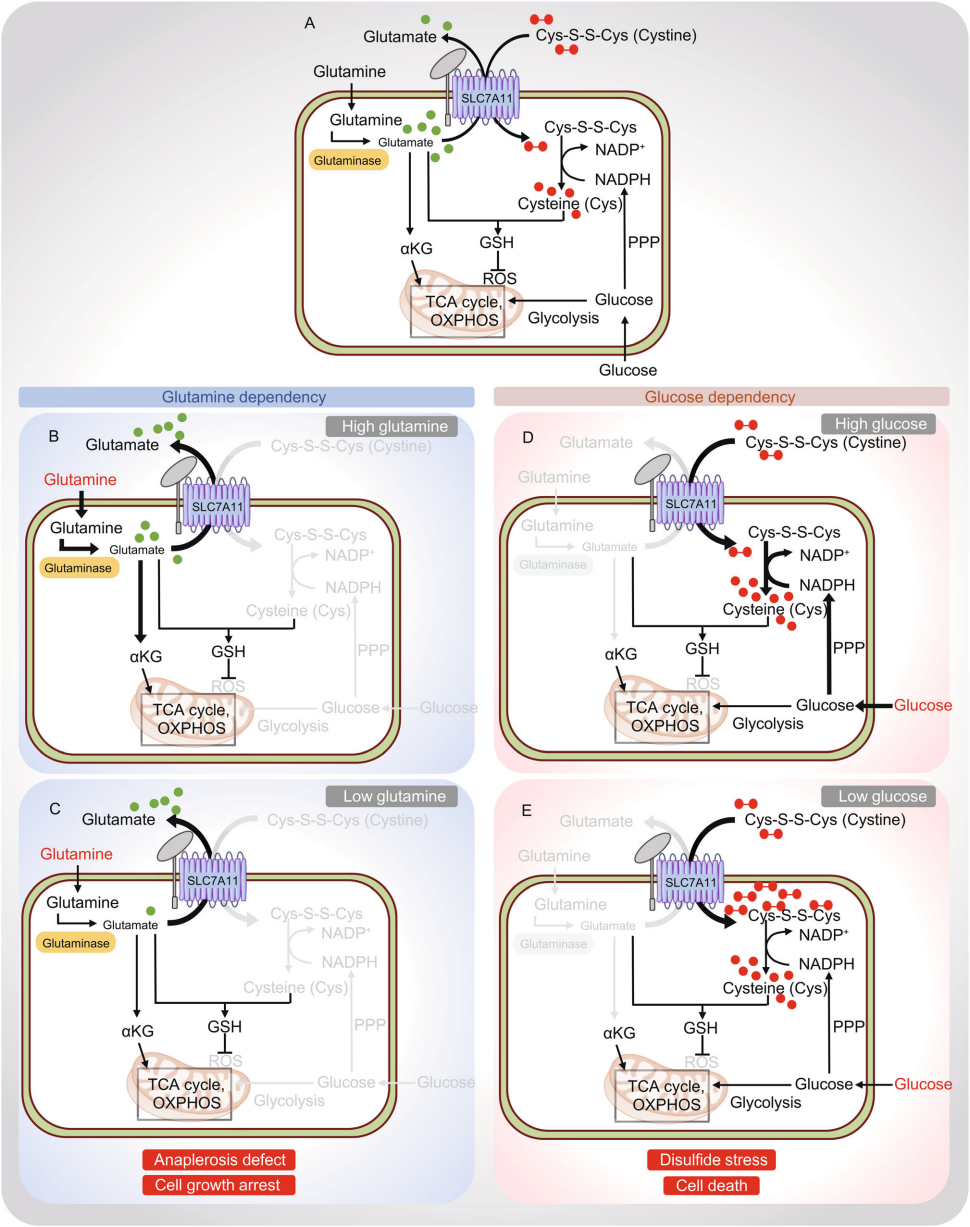
**SLC7A11 regulates glucose and glutamine dependencies in cancer cells**. (A) SLC7A11 functions to import extracellular cystine in exchange for intracellular glutamate. Extracellular glutamine is imported into the cell and converted to glutamate by Glutaminase. Subsequently, glutamate serves to fuel the TCA cycle via its conversion to α-ketoglutarate and acts as a precursor for GSH synthesis. The other arm of SLC7A11 transporter function imports extracellular cystine. Once imported into the cell, cystine is reduced to cysteine by the consumption of NADPH. Cysteine then serves as the rate limiting precursor for GSH synthesis, and GSH suppresses ROS. NADPH is mainly supplied from glucose via the PPP. Consequently, an imbalance in this metabolic network leads to specific metabolic dependencies in SLC7A11^high^ cancer cells. (B and C) *Glutamine dependency*: In SLC7A11^high^ cancer cells, increased glutamate export results in a partial depletion of intracellular glutamate that can feed into the TCA cycle. This pushes SLC7A11^high^ cancer cells to uptake more glutamine and activates glutaminase to promote glutamine conversion to glutamate, resulting in glutamine dependency (B). When SLC7A11^high^ cancer cells (with increased glutamate export) are challenged with low glutamine availability, intracellular glutamate is inadequate to fuel the TCA cycle, resulting in anaplesrosis defect and cell growth arrest (C). (D and E) *Glucose dependency*: In SLC7A11^high^ cancer cells, large amount of extracellular cystine is imported into the cell. Due to its low solubility, buildup of intracellular cystine is likely toxic, forcing cells to quickly reducing cystine to much more soluble cysteine in the cytosol. Because this reaction requires NADPH and cytosolic NADPH is mainly supplied from glucose via the PPP, SLC7A11^high^ cancer cells exhibit glucose-PPP dependency. (D) Limiting glucose availability in SLC7A11^high^ cancer cells leads to NADPH depletion, accumulation of intracellular cystine and other disulfide molecules resulting in disulfide stress and rapid cell death (E). GSH: reduced glutathione, PPP: pentose phosphate pathway, TCA cycle: tricarboxylic acid cycle, αKG: α-ketoglutarate

As extensively discussed in previous sections, SLC7A11-mediated cystine uptake is critical for cancer cells to suppress ferroptosis and to maintain redox homeostasis and biomass incorporation. Many cancer cells upregulate SLC7A11 levels to enhance their capability to obtain extracellular cystine. Emerging evidence indicates that, however, this comes at a significant cost for SLC7A11^high^ cancer cells. In our view, these “costs” are mainly derived from two metabolic features associated with cystine uptake mediated by SLC7A11. First, as an antiporter, SLC7A11-mediated cystine uptake is coupled with glutamate export (Fig. 4A). Consequently, a significant amount of intracellular glutamate is exported out of cells in exchange for cystine uptake in SLC7A11^high^ cancer cells. Indeed, it was estimated that 30%–50% of intracellular glutamate is exported by SLC7A11 in exchange for extracellular cystine (Bannai and Ishii, 1988). In addition, due to the oxidizing extracellular environment, extracellular cystine is much more stable than extracellular cysteine, resulting in a much higher concentration of extracellular cystine than that of extracellular cysteine. In order to obtain cysteine, cancer cells primarily rely on taking up extracellular cystine (rather than extracellular cysteine) and then need to convert intracellular cystine to cysteine (Fig. 4A) (this feature is unique in nutrient uptake. For most other nutrients, such as glucose and glutamine, cells can directly take up the nutrients they require through corresponding nutrient transporters). Because the reduction of cystine to cysteine consumes NADPH, cells need to first make redox “investment” (in the form of NADPH) in order to obtain cysteine for maintaining redox homeostasis [which is somewhat similar to “ATP investment” made in glycolysis, wherein two molecules of ATP are “invested” (i.e., consumed) in the first several steps of glycolysis in order to activate glucose for its subsequent generation of four molecules of ATPs in the later steps of glycolysis]. Consequently, these two “costs” associated with SLC7A11-mediated cystine uptake, namely glutamate export and NADPH consumption for cystine reduction, drive SLC7A11^high^ cancer cells to be highly dependent on glutamine and glucose. In this section, we will discuss recent studies leading to our current understanding of SLC7A11-induced glutamine and glucose dependency.

### SLC7A11-induced glutamine dependency in cancer cells

Glutamine is a critical amino acid that supports bioenergetic and biosynthetic processes and maintains redox balance in cancer cells (Hensley et al., 2013). It is also the most abundant amino acid in plasma as well as in cell culture media. Once imported into cells via SLC1A5 and other transporters, glutamine is converted to glutamate via glutaminase. Glutamate has several important functions in cellular metabolism, prominent among which are its roles in anaplerosis [replenishing the tricarboxylic acid (TCA) cycle through its conversion to TCA cycle intermediate α-ketoglutarate (αKG)] and as a precursor for GSH synthesis (Fig. 4A) (Hensley et al., 2013). As noted above, glutamate is also exported out of cells via SLC7A11 in exchange for extracellular cystine. In SLC7A11^high^ cancer cells, high glutamate export results in a partial depletion of intracellular glutamate, which then drives cells through the relief of feedback inhibition to take up more glutamine as well as to activate glutaminase for glutamate replenishment, leading to glutamine dependency in SLC7A11^high^ cancer cells (Fig. 4B). Consequently, SLC7A11^high^ cancer cells are more sensitive to glutamine starvation (Fig. 4C).

This model has been supported by several studies in recent years. An analysis of glutamine uptake and dependency across a wide range of breast cancer cells revealed that, compared to normal mammary epithelial cells and basal breast cancer cells, most basal and claudin-low triple-negative breast cancer (TNBC) cells consume more glutamine and exhibit more glutamine dependency (Timmerman et al., 2013). Differential glutamine dependency among these breast cancer cells does not correlate with the expression levels of known regulators in glutamine metabolism, such as glutaminase; instead, it was shown that the sensitivities of these cells to glutamine deprivation correlate with SLC7A11 expression levels and cystine consumption rates (i.e., compared to other breast cancer cell lines, basal and claudin-low TNBC cells express higher levels of SLC7A11, exhibit more cystine consumption, and are more sensitive to glutamine deprivation) (Timmerman et al., 2013). These data suggest that the glutamine dependency phenotype observed in these TNBC cells is likely driven by the high expression of SLC7A11 and the high rate of SLC7A11-mediated cystine uptake in these cells.

In another study aimed to identify the environmental factors that underlie the less glutamine dependence of tumor cells grown *in vivo* than those grown *in vitro*, cystine was identified as the sole factor that causes the higher glutamine dependency for cancer cells cultured in standard cell culture media than the same cells cultured in media that better mimic *in vivo* conditions; therefore, compared to *in vivo* conditions, higher levels of cystine in standard cell culture media drive more SLC7A11-mediated cystine uptake and glutamate export, resulting in more glutamine dependency and more cellular sensitivity to glutaminase inhibition (Muir et al., 2017). As discussed in a previous section, *SLC7A11* transcription is controlled by the KEAP1-NRF2 signaling axis. In line with this, it was shown that cancer cells with *KEAP1* mutation and/or NRF2 hyperactivation exhibit increased glutamine dependency and enhanced sensitivity to glutamine deprivation or glutaminase inhibition, partly via high SLC7A11-mediated glutamate export in these cancer cells (Romero et al., 2017; Sayin et al., 2017).

### SLC7A11-induced glucose dependency in cancer cells

One hallmark of metabolic reprogramming in cancer cells is the deregulated uptake of glucose (Pavlova and Thompson, 2016). Upon its transport into cells through glucose transporters (GLUTs), glucose is subsequently shunted into two major metabolic pathways, glycolysis and the pentose phosphate pathway (PPP) (Vander Heiden et al., 2009). Via glycolysis, glucose is metabolized to pyruvate, which can be oxidized by the TCA cycle to generate ATP. By contrast, the PPP uses glucose to generate both ribose-5-phosphate, for nucleic acid synthesis, and NADPH, which provides the reducing power to support reductive biosynthetic reactions and to maintain cellular redox homeostasis.

Glucose is one of the principal nutrients supporting cancer cell survival (Vander Heiden et al., 2009; Boroughs and DeBerardinis, 2015). Several studies independently revealed that SLC7A11^high^ cancer cells are highly dependent on glucose for survival and are exquisitely sensitive to glucose starvation-induced cell death (Goji et al., 2017; Koppula et al., 2017; Shin et al., 2017; Liu et al., 2020). In one study, loss-of-function screens uncovered both SLC7A11 and SLC3A2 as the top genes whose inactivation promotes cell survival under glucose starvation (Shin et al., 2017). In another study, SLC7A11 expression was identified to be highly induced upon glucose starvation (Koppula et al., 2017). As discussed in previous sections, stress-induced SLC7A11 expression generally serves as an adaptive response to protect cells from stress-induced cell death (such as ferroptosis); therefore, it was initially hypothesized that glucose starvation-induced SLC7A11 expression might protect cells from cell death triggered by glucose starvation. Surprisingly, it was subsequently shown that SLC7A11 overexpression drastically promotes glucose starvation-induced cell death, whereas SLC7A11 inactivation significantly suppresses cell death under glucose starvation (Koppula et al., 2017; Shin et al., 2017). Together, these studies revealed that SLC7A11 promotes glucose dependency in cancer cells.

While it is easy to understand the metabolic underpinning of SLC7A11-induced glutamine dependency (given that SLC7A11-mediated glutamate export is directly linked to glutamine metabolism), how SLC7A11 promotes glucose dependency has remained less clear (partly because SLC7A11-associated metabolism is not directly connected to glucose metabolism). Since both glucose and glutamate are important for TCA cycle anaplerosis and mitochondrial respiration, it was initially proposed that, because SLC7A11-mediated glutamate export decreases intracellular glutamate reserves, SLC7A11^high^ cancer cells are forced to have more reliance on glucose to replenish the TCA cycle (presumably through glycolysis) and to maintain mitochondrial respiration, resulting in glucose dependency (Koppula et al., 2017; Shin et al., 2017). In support of this, it was shown that supplementation of αKG, a TCA cycle intermediate derived from glutamate, rescues glucose starvation-induced cell death in SLC7A11^high^ cancer cells (Koppula et al., 2017; Shin et al., 2017). However, other recent data argued against this model. For example, this model would predict that SLC7A11 overexpression should promote glycolytic flux and that SLC7A11^high^ cancer cells should be sensitive to glycolysis inhibitors. However, a recent study showed that SLC7A11 overexpression does not promote glycolytic flux and that 2-deoxyglucose (2DG), a glycolysis inhibitor, even rescues glucose starvation-induced cell death in SLC7A11^high^ cancer cells (the underlying mechanism will be described below) (Koppula et al., 2017; Shin et al., 2017). Furthermore, it was shown that cystine starvation rescues the cell death in SLC7A11^high^ cancer cells under glucose starvation, suggesting that it is cystine import that underlies glucose starvation-induced cell death in SLC7A11^high^ cancer cells (Goji et al., 2017; Joly et al., 2020; Liu et al., 2020).

Mechanistically, it was proposed that, because cystine is highly insoluble, significant buildup of intracellular cystine potentially is toxic to cells, forcing SLC7A11^high^ cancer cells to quickly reduce cystine to much more soluble cysteine once cystine is taken up into cells. Since cystine reduction to cysteine consumes NADPH, this renders SLC7A11^high^ cancer cells to be highly dependent on the glucose-PPP route (which is the major provider of cellular NADPH) to supply NADPH for cystine reduction (Fig. 4D). Consequently, glucose starvation limits NADPH supply, resulting in NADPH depletion, marked accumulation of cystine and other disulfide molecules, ROS accumulation, and rapid cell death (Fig. 4E). It should be noted that the fold changes of these redox defects are much more pronounced than that of glutamate level decrease in SLC7A11^high^ cancer cells under glucose starvation, and thereby correlate better with the rapid and dramatic cell death observed in these cells upon glucose starvation (Liu et al., 2020). In further support of this model, glucose starvation-induced cell death and redox defects in SLC7A11^high^ cancer cells can be substantially blocked by treatments that restore NADPH levels and prevent cystine accumulation, such as treatment with 2DG (which inhibits glycolysis but can still be shunted into the PPP to generate NADPH) (Liu et al., 2020), suggesting that it is cystine accumulation and/or NADPH depletion resulting from cystine uptake that causes the cell death in SLC7A11^high^ cancer cells under glucose starvation.

There are several peculiar features associated with SLC7A11-induced glucose dependency that are worth further discussion. Because SLC7A11 has a well-established role in suppressing ROS under oxidative stress conditions, the observation that SLC7A11 overexpression dramatically increases ROS levels (Goji et al., 2017; Liu et al., 2020) under glucose starvation is rather counterintuitive. Earlier, we introduced the concept that cells need to first make redox “investment” (by supplying NADPH to reduce cystine to cysteine) in order to obtain cysteine for its subsequent utilization in redox maintenance. These data suggest that the failure in appropriately maintaining this redox “investment” (such as under glucose starvation) can have devastating cellular consequences by “bankrupting” the redox system in SLC7A11^high^ cancer cells—somewhat analogous to company bankruptcy resulting from finance investment mismanagement. In addition, as discussed in previous sections, cystine in culture media is generally required for cell survival and cystine starvation is well known to induce ferroptosis; therefore, the observation that cystine starvation also rescues glucose starvation-induced cell death in SLC7A11^high^ cancer cells seems confusing. It should be noted that glucose starvation-induced cell death in SLC7A11^high^ cancer cells is not ferroptosis (Liu et al., 2020) and that SLC7A11^high^ cancer cells have high intracellular cysteine reserves and therefore are generally resistant to cystine starvation (Zhang et al., 2018b; Liu et al., 2020). Consequently, at the time points when cystine starvation has not induced obvious ferroptosis in SLC7A11^high^ cancer cells, it actually prevents glucose starvation-induced cell death in these cells. In other words, in SLC7A11^high^ cancer cells, glucose starvation induces far more acute cellular toxicity than cystine starvation.

Taken together, these studies suggest that SLC7A11 overexpression induces both glutamine and glucose dependency in cancer cells with different underlying mechanisms. While SLC7A11-induced glutamine dependency mainly results from glutamate-derived anaplerosis (Fig. 4B), it seems that SLC7A11-induced glucose dependency largely relates to cystine-associated redox maintenance (although glutamate-derived anaplerosis might still play a role in it) (Fig. 4D). Correspondingly, the cellular phenotypes in SLC7A11^high^ cancer cells in response to glutamine or glucose deprivation seem somewhat different: while glutamine deprivation (or glutaminase inhibition) mainly suppresses the cell growth in SLC7A11^high^ cancer cells (which is in line with anaplerosis defects in these cells) (Romero et al., 2017) (Fig. 4C), glucose starvation induces rapid cell death in SLC7A11^high^ cancer cells (consistent with drastic redox system collapse in this context) (Goji et al., 2017; Koppula et al., 2017; Shin et al., 2017; Liu et al., 2020) (Fig. 4E). In further support of this, N-acetyl cysteine (NAC) largely rescues cell death in SLC7A11^high^ cancer cells under glucose starvation (Liu et al., 2020), but does not rescue the growth defect of these cells upon glutamine deprivation or glutaminase inhibition (Romero et al., 2017). In the next section, we will further discuss how our mechanistic understanding of SLC7A11-induced nutrient dependency can help inform therapeutic strategies to target SLC7A11 in cancer.

## THERAPEUTIC STRATEGIES TARGETING SLC7A11 IN CANCER

In recent years, SLC7A11 has emerged as a promising therapeutic target in cancer therapy. To achieve appropriate therapeutic index in cancer therapy, the ideal therapeutic target being inhibited by cancer drugs presumably should have a selective role in tumor growth with a dispensable function in normal physiology, so that the corresponding drugs can produce the desired toxic effects in cancer cells (or tumors) without unwanted side effects in normal cells (or normal tissues). This seems to be the case for SLC7A11. *Slc7a11* KO mice are viable, with no obvious phenotype in the major organs (Sato et al., 2005). Subsequent analyses revealed defects in spatial working memory and skin pigmentation in *Slc7a11* KO or mutant mice (Chintala et al., 2005; De Bundel et al., 2011); nonetheless, these phenotypes are relatively minor. Considering the essential role of SLC7A11 in defending ferroptosis, the mild phenotype from *Slc7a11* KO mice may seem somewhat surprising (as a comparison, germline or postnatal deletion of *Gpx4*, another essential anti-ferroptosis regulator, results in embryonic or adult lethality, respectively (Yant et al., 2003; Friedmann Angeli et al., 2014)). It is possible that normal cells or tissues can compensate for the loss of *SLC7A11* by obtaining intracellular cysteine through *de novo* cysteine synthesis or cystine (or cysteine) uptake via additional transporters (such as SLC7A9-mediated cystine uptake in the kidney) (Stipanuk, 2004; Kandasamy et al., 2018). Because cancer cells often experience high levels of oxidative stress (Chio and Tuveson, 2017), they have significantly increased needs in antioxidant defense and therefore are much more dependent on SLC7A11-mediated cystine uptake to obtain cysteine and to maintain redox homeostasis than are normal tissues—much analogous to oncogene addiction in cancer development. Consistent with this, SLC7A11 is overexpressed in multiple cancer types, including lung cancer, TNBC, PDAC, renal cell carcinoma, liver cancer, and glioma, and its high expression often correlates with poor prognosis (Timmerman et al., 2013; Ji et al., 2018; Koppula et al., 2018; Zhang et al., 2018a; Zhang et al., 2018b; Badgley et al., 2020). In further support of this, deletion of *Slc7a11* in adult mice does not affect normal pancreas development but significantly impairs Kras-driven PDAC development (Badgley et al., 2020).

**Figure 5:**
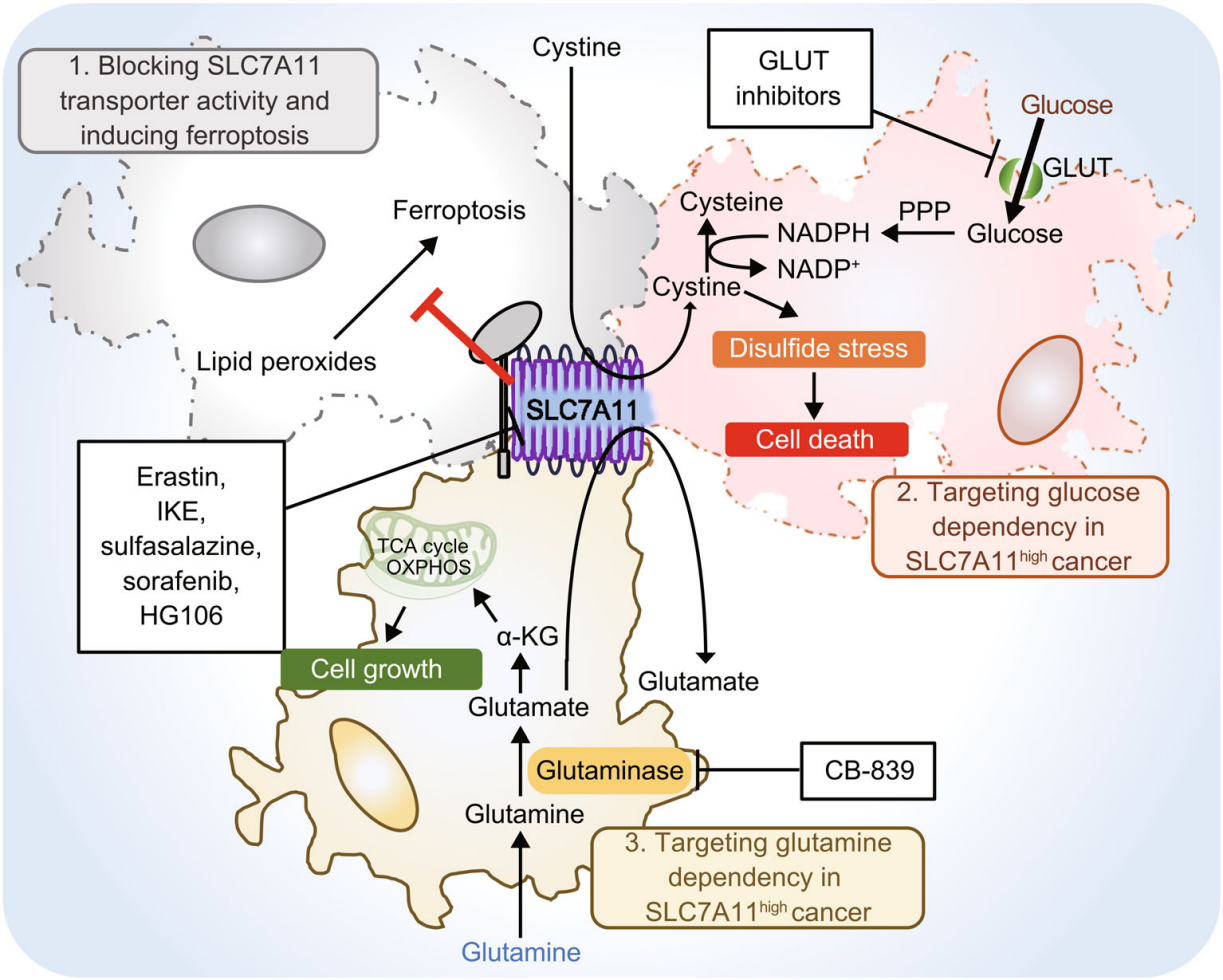
**Therapeutic strategies to target SLC7A11 in cancer**. The antiporter function of SLC7A11 offers several strategies for therapeutic targeting. (1) Direct blocking of SLC7A11 cystine transporter activity using its inhibitors such as erastin, IKE, sulfasalazine, sorafenib, and HG106. These drugs inhibit cystine uptake by SLC7A11, thereby inducing lipid peroxidation and ferroptotic cell death. (2) Targeting glucose dependency in SLC7A11^high^ cancer cells by inhibiting glucose uptake using GLUT inhibitors. Decreased glucose availability in in SLC7A11^high^ cancer cells induce disulfide stress, leading to rapid cell death. (3) Targeting glutamine dependency in SLC7A11^high^ cancer cells by using glutaminase inhibitors such as CB-839. Glutaminase inhibition decreases glutamate-derived anaplerosis and induces cell growth arrest in SLC7A11^high^ cancer cells. IKE: imidazole ketone erastin, GLUT: glucose transporter

These two characteristics of SLC7A11, namely its dispensability in normal physiology and its high expression in cancers, suggest that targeting SLC7A11 likely can selectively kill tumor cells and impair tumor growth while sparing normal cells or tissues, therefore nominating SLC7A11 as a promising therapeutic target in cancer treatment. Below, we further discuss recent preclinical studies aimed to target SLC7A11 in cancer. To help readers better understand these works, we have conceptualized these studies into two broad strategies (Fig. 5). The first involves directly inhibiting SLC7A11 transporter activity, whereas the other relates to targeting SLC7A11-associated metabolic vulnerabilities (glucose or glutamine dependency) in cancer. (To make an analogy here, these strategies are similar to those used to target KRAS-driven cancers: while some investigators have focused on directly inhibiting oncogenic KRAS, with the recently developed Kras (G12C) inhibitor AMG510 as a notable example (Canon et al., 2019), others have aimed instead to target KRAS-associated vulnerabilities, for example, the efforts to identify synthetically lethal interactions with KRAS (Cox et al., 2014)).

Several compounds have been identified or characterized as SLC7A11 inhibitors, including erastin, imidazole ketone erastin (IKE), sulfasalazine, sorafenib, and HG106 (Feng and Stockwell, 2018; Hu et al., 2020). Given their capabilities of inducing ferroptosis through blocking SLC7A11-mediated cystine uptake, erastin, IKE, sulfasalazine, and sorafenib are also collectively called class 1 ferroptosis inducers (FINs) in ferroptosis studies. From the therapeutic point of view, each of these compounds has its pros and cons. Erastin probably is the most widely used SLC7A11 inhibitor or class 1 FIN in cell culture studies; however, due to its poor metabolic stability and low solubility *in vivo*, erastin cannot be used for animal studies (Feng and Stockwell, 2018), preventing its further testing in preclinical or clinical studies. IKE is an erastin analog with nanomolar potency and high metabolic stability that is suitable for animal treatment (Zhang et al., 2019b). Recent studies using GEMMs also demonstrated its efficacy in suppressing Kras-driven PDAC development (Badgley et al., 2020). However, because IKE was developed relatively recently, it has not moved to the clinical trial stage yet. Sorafenib and sulfasalazine, in contrast, are drugs that were already approved by the U.S. Food and Drug Administration (FDA). Previous studies showed that both drugs inhibit SLC7A11 transporter activity, and can induce ferroptosis and suppress tumor growth *in vivo* (Feng and Stockwell, 2018; Lei et al., 2020; Ye et al., 2020). However, both drugs have mechanisms of action besides inhibiting SLC7A11 and inducing ferroptosis: sorafenib is commonly used in cancer therapy as a multi-kinase inhibitor, whereas sulfasalazine is capable of blocking prostaglandin production and is commonly used for treating patients with arthritis. Therefore, it is critical to determine whether their anti-cancer effect under any given context is indeed caused by SLC7A11 inhibition and ferroptosis induction. Finally, although HG106 was recently identified as a SLC7A11 inhibitor (Hu et al., 2020), whether it can act as a FIN remains to be further examined (so far, all other SLC7A11 inhibitors can induce ferroptosis). Together, although preclinical studies have established the proof of concept to inhibit SLC7A11 in cancer therapy, there is still a significant need to further identify potent and specific SLC7A11 inhibitors, study their mechanisms of action, test them in rigorous preclinical models, and eventually apply them in clinical care.

As another approach, rather than directly inhibiting SLC7A11, various studies have tested targeting metabolic vulnerabilities associated with SLC7A11^high^ cancers. As discussed in the preceding section, SLC7A11^high^ cancer cells are dependent on glutamine for glutamate-derived anaplerosis, which prompted the hypothesis that SLC7A11^high^ cancer cells or tumors may be particularly sensitive to glutaminase inhibition. *KEAP1* is frequently mutated in lung cancer, and *KEAP1*-mutant lung cancer exhibits NRF2 hyperactivation and aberrant expression of SLC7A11 (Cancer Genome Atlas Research, 2012, 2014). Consistent with this, it was shown that glutaminase inhibitor CB-839 suppresses tumor growth of *KEAP1*-mutant cell line- or patient-derived xenografts (PDXs) much more potently than that of *KEAP1*-WT counterparts (Romero et al., 2017; Galan-Cobo et al., 2019). In the previous section, we also discussed glucose dependency of SLC7A11^high^ cancer cells; consequently, SLC7A11^high^ cancer cells are exquisitely sensitive to glucose starvation-induced cell death. Likewise, compared with SLC7A11^low^ cancer cells, SLC7A11^high^ cancer cells exhibit much more vulnerability to GLUT inhibitor-induced cell death (Liu et al., 2020). It was further demonstrated that KL-11743, a potent GLUT1/3 dual inhibitor, selectively suppresses SLC7A11^high^ tumor growth in both cell line xenografts and PDXs (Liu et al., 2020). These studies together suggest the possibility that SLC7A11 expression can be used as a biomarker to select cancer patients for glutaminase or GLUT inhibitor treatment.

## CONCLUSIONS AND FUTURE PERSPECTIVES

Cancer cells upregulate SLC7A11 expression through diverse mechanisms to enhance their antioxidant defense and to suppress ferroptosis (a tumor suppression mechanism), which is beneficial for tumor growth. However, emerging evidence suggests that building up cysteine-derived antioxidant defense systems also comes at an enormous cost for SLC7A11^high^ cancer, as such efforts require a significant “investment” of cellular resources, including glutamate export for exchange of cystine import and NADPH supply to reduce cystine to cysteine in the cytosol, resulting in glucose- and glutamine-dependency in SLC7A11^high^ cancer cells. Because glucose and glutamine are among the most abundant nutrients in the extracellular environment, under normal conditions, SLC7A11^high^ cancer cells can take up sufficient amounts of glucose and glutamine to meet these demands for their antioxidant defense. On the basis of this, we propose that under most conditions, SLC7A11 primarily exerts a tumor promoting effect, but its overexpression does expose an Achilles’ heel in cancer cells (by rendering them to be more sensitive to glucose or glutamine starvation). We further reason that under glucose or glutamine limiting conditions, SLC7A11 may even exert a tumor-suppressive effect, a hypothesis remaining to be tested. Below we highlight a few other key questions for further understanding and targeting SLC7A11 in tumor biology in future studies.

Until relatively recently, only NRF2 and ATF4 were known to regulate *SLC7A11* transcription. In the past two years, we have witnessed a flurry of findings revealing transcriptional regulation of SLC7A11 by diverse transcription factors and epigenetic regulators (Fig. 2). As such, we now have a much deeper understanding of SLC7A11 regulation at the transcriptional level. In contrast, our knowledge of SLC7A11 regulation at the posttranslational level remains relatively rudimentary. In addition, how such posttranslational regulations in turn modulate SLC7A11-mediated downstream biological effects, such as ferroptosis and nutrient dependency, still remains largely unknown. For example, considering important roles of mTORC2 and AKT in promoting cell survival (Manning and Toker, 2017), the findings that mTORC2- or AKT-mediated phosphorylation of SLC7A11 inhibits SLC7A11’s transporter activity may seem somewhat counterintuitive (Gu et al., 2017; Lien et al., 2017). Since SLC7A11 promotes cell death under glucose starvation, it will be interesting to test whether this inhibitory phosphorylation serves to suppress SLC7A11 function in response to glucose starvation, thereby promoting cell survival under glucose limiting conditions. Further studies are needed to further understand these intriguing questions.

Further, considering SLC7A11’s function to import cystine from and export glutamate into extracellular environment, SLC7A11 might play a role in mediating the cross-talk between tumor cells and tumor microenvironment. However, most current studies have focused on the cell autonomous function of SLC7A11 in tumor cells. We envision that future studies will elucidate potential cell non-autonomous functions of SLC7A11 in tumor biology. Likewise, whether and how SLC7A11 function in immune cells or stromal cells in turn modulates tumor cell behavior is also a potentially fascinating topic for future investigation.

The identity of the cystine reductase(s) that consume NADPH to reduce cystine to cysteine still remains elusive. Previous studies implicated that thioredoxin-related protein of 14 kDa (TRP14) and thioredoxin reductase 1 (Txnrd1) are potential cystine reductases (Mandal et al., 2010; Pader et al., 2014). Both TRP14 and Txnrd1 are indeed coupled to NADPH. However, further genetic studies are required to establish them as bona fide cystine reductases (for example, to show that inactivation of the presumed cystine reductase can dramatically increase intracellular cystine levels).

In this review, we summarized two broad strategies to target SLC7A11; one is to directly inhibit SLC7A11-mediated cystine uptake, the other is to target SLC7A11-induced glucose or glutamine dependency (Fig. 5). To move these findings to clinic application, we propose that two questions should be further addressed in future preclinical studies. The first is to identify the specific tumor or genotype context for therapeutic targeting. Based on the recent preclinical studies (Romero et al., 2017; Liu et al., 2020), it is clear that SLC7A11^high^ tumors should be selected for GLUT or glutaminase inhibitors (to target glucose or glutamine dependency, respectively). Since *KEAP1* or *BAP1* mutant cancers exhibit high SLC7A11 expression (Cancer Genome Atlas Research, 2012, 2014; Zhang et al., 2018b), *KEAP1* or *BAP1* mutation can be used as potential biomarkers to select SLC7A11^high^ tumors for GLUT or glutaminase inhibition. On the other hand, it remains unclear whether SLC7A11^high^ or SLC7A11^low^ tumors should be selected for SLC7A11 inhibitor treatment. Presumably, SLC7A11^low^ cancer cells should be sensitive to SLC7A11 inhibition. However, it is also possible that SLC7A11^high^ cancer cells are particularly dependent on SLC7A11, therefore rendering SLC7A11^high^ cancer to be sensitive to SLC7A11 inhibitors too. In addition, it has been shown that tumors with certain oncogenic mutations (such as *KRAS* mutant tumors (Daher et al., 2019; Lim et al., 2019; Badgley et al., 2020; Hu et al., 2020)) are dependent on SLC7A11-mediated cystine uptake, and therefore such tumors could be particularly sensitive to SLC7A11 inhibitors. Future studies should be directed to further test these hypotheses.

The second question is to identify rational combination strategies with these inhibitors for therapeutic targeting. In this regard, recent studies uncovered that immunotherapy and radiotherapy, two commonly used cancer therapies, can potently induce ferroptosis in cancer cells; further preclinical analyses revealed potent therapeutic efficacy to combine immunotherapy (or radiotherapy) with SLC7A11 inhibitors (IKE, sulfasalazine, sorafenib; class 1 FINs) (Lang et al., 2019; Wang et al., 2019a; Lei et al., 2020; Ye et al., 2020). These studies provide strong justifications to combine these standard-of-cares with SLC7A11 inhibitors in cancer therapy. The rational combination therapies with GLUT or glutaminase inhibitors to treat SLC7A11^high^ cancer remain to be established.
